# Identification of Candidate Genes and eQTLs Related to Porcine Reproductive Function

**DOI:** 10.3390/ani15071038

**Published:** 2025-04-03

**Authors:** Tong Zeng, Ji Wang, Zhexi Liu, Xiaofeng Wang, Han Zhang, Xiaohua Ai, Xuemei Deng, Keliang Wu

**Affiliations:** 1 National Engineering Laboratory for Animal Breeding, Department of Animal Genetics and Breeding, College of Animal Science and Technology, China Agricultural University, Beijing 100193, China; woshizttttt@163.com (T.Z.); ji.wang@cau.edu.cn (J.W.); 15928144217@163.com (Z.L.); hanzhang@cau.edu.cn (H.Z.); aliceaxh@163.com (X.A.); 2 Frontier Technology Research Institute of China Agricultural University in Shenzhen, Shenzhen 518119, China; 3 Beijing Municipal General Station for Animal Husbandry & Veterinary Service, Beijing 100107, China; wangxf@126.com; 4 Sichuan Advanced Agricultural & Industrial Institute, China Agricultural University, Chengdu 611430, China

**Keywords:** eQTL, WGCNA, endometrium, reproductive function, pigs

## Abstract

Expression quantitative trait locus (eQTL) mapping is an effective tool for identifying genetic variations that regulate gene expression. An increasing number of studies suggested that SNPs associated with complex traits in farm animals are considered as expression quantitative trait loci. Identifying eQTLs associated with gene expression levels in the endometrium helps to unravel the regulatory mechanisms of genes related to reproductive functions in this tissue and provides molecular markers for the genetic improvement of high-fertility sow breeding. In this study, 218 RNA-seq data from pig endometrial tissue were used for eQTL analysis to identify genetic variants regulating gene expression. Additionally, weighted gene co-expression network analysis (WGCNA) was performed to identify hub genes involved in reproductive functions. The eQTL analysis identified 34,876 significant cis-eQTLs regulating the expression of 5632 genes (FDR ≤ 0.05), and 90 hub genes were identified by WGCNA analysis. By integrating eQTL and WGCNA results, 14 candidate genes and 16 fine-mapped cis-eQTLs were identified, including *FRK*, *ARMC3*, *SLC35F3*, *TMEM72*, *FFAR4*, *SOWAHA*, *PSPH*, *FMO5*, *HPN*, *FUT2*, *RAP1GAP*, *C6orf52*, *SEL1L3*, and *CLGN*, which were involved in the physiological processes of reproduction in sows through hormone regulation, cell adhesion, and amino acid and lipid metabolism. These eQTLs regulate the high expression of candidate genes in the endometrium, thereby affecting reproductive-related physiological functions. These findings enhance our understanding of the genetic basis of reproductive traits and provide valuable genetic markers for marker-assisted selection (MAS), which can be applied to improve sow fecundity and optimize breeding strategies for high reproductive performance.

## Simple Summary

Reproductive traits are important economic traits in pigs, and the endometrium is closely associated with reproductive traits such as litter size. In this study, the eQTL and WGCNA analysis integrated RNA-seq data of pig endometrium were performed to identify candidate genes and eQTLs related to pig reproductive functions. These findings provide a theoretical foundation for improving reproductive performance in pigs.

## 1. Introduction

Reproductive traits of sows, including total number born (TNB), number of piglets born alive (NBA), litter birth weight (LBW), and gestation length (GL), are important economic traits that affect pig production efficiency [1], and improving the reproductive performance of sows has become an important task in modern livestock farming. Due to the low heritability of reproductive traits (approximately 0.1–0.3) and their regulation by multiple genes, it is difficult to improve these traits faster using traditional breeding methods [2,3]. With the development of molecular breeding techniques, marker-assisted selection (MAS) and genomic selection (GS) have become effective ways to improve sow reproductive performance [4,5].

In the reproductive process of sows, the endometrium plays a key role in embryo implantation, pregnancy maintenance, and fetal development [6]. The function of the endometrium is regulated by a complex interplay of hormones, signaling molecules, and gene expression, and its status directly affects the reproductive performance of sows [7,8]. In previous studies, our group identified *BMPR1B* as a candidate gene influencing litter size in Taihu pigs through whole-genome resequencing. We found that an estrogen receptor ESR1 response element (ERE) exists in the first intron of *BMPR1B*, which can cis-regulate its expression, thereby affecting the development of endometrial glands in pregnant sows and increasing litter size by 0.4 to 1 piglet [9,10,11]. This study demonstrates that genetic variation can influence reproductive traits by regulating gene expression, highlighting the importance of understanding the genetic regulatory mechanisms of gene expression in revealing the genetic basis of reproductive traits.

Currently, genome-wide association studies (GWAS) and quantitative trait locus (QTL) studies have identified several genetic variations and candidate genes associated with reproductive traits, providing a theoretical basis for marker-assisted selection [12,13,14]. Since GWAS relies on statistical associations between genetic loci and phenotypes, its results are limited in directly revealing the biological functions of genetic loci, especially as most associated variants are located in non-coding regions, and their mechanisms remain unclear [15]. Therefore, understanding how genetic variation affects gene expression and subsequently regulates complex traits is an important direction for current research.

eQTL mapping is a powerful tool for elucidating the genetic regulation of gene expression, which reveals the key loci that regulate gene expression by identifying the association between gene expression levels and genetic variation [16]. Within the framework of the central law of molecular biology, eQTLs can influence gene expression through multiple mechanisms, including transcriptional regulation, epigenetic modifications, and post-transcriptional regulation [17]. At the level of transcriptional regulation and epigenetic modification, eQTLs can influence processes such as DNA methylation, histone modifications, enhancer activity, and transcription factor binding, thereby altering transcription initiation and making major contributions to variation in steady-state mRNA levels [18]. Due to the high tissue specificity of gene expression in eukaryotes, different tissues exhibit distinct gene expression patterns [19]. Therefore, it is essential to investigate gene regulatory mechanisms within specific tissues. Previous studies have demonstrated that SNPs associated with complex traits in farm animals are considered expression quantitative trait loci (eQTLs) [20,21]. For example, in boars, a cis-eQTL (rs339380065) for the *CYP1A2* gene in testicular tissue may be a key marker influencing boar taint (an animal welfare trait) [22]. Therefore, eQTL mapping is valuable in resolving the genetic basis of reproductive traits. However, reproductive traits, especially litter size, are intricately regulated by gene expression in the sow endometrium, and the specific mechanisms by which genetic variants in the endometrium regulate the expression of relevant genes to influence its reproductive function remain unclear.

To further elucidate these genetic regulatory mechanisms, this study conducted an eQTL analysis using porcine endometrial tissue. Since pig endometrial tissues are difficult to obtain in large quantities, integrating resources from public databases is an effective approach to performing eQTL analysis, with reference to the methods of the Genotype-Tissue Expression (GTEx) project [23] and the Farm Animal Genotype-Tissue Expression (FarmGTEx) project [24]. While we referenced the eQTL research methods of the FarmGTEx project, we specifically incorporated a WGCNA analysis approach, focusing on the reproductive biological functions of porcine endometrial tissue, which has not been explored previously. Therefore, this study integrates raw RNA-seq data from both public datasets and our research group, identifying eQTLs and candidate genes influencing porcine reproductive function through eQTL analysis and WGCNA. This provides new insights into the genetic basis of pig reproductive processes and offers molecular markers for improving sow reproductive performance.

## 2. Materials and Methods

### 2.1. Data Sources

In this study, we used 341 RNA-seq data (218 from endometrial samples, 78 from ovarian samples, and 45 from placental samples), including 326 RNA-seq data downloaded from the Sequence Read Archive and European Nucleotide Archive (Supplementary Table S1), and 25 RNA-seq data (15 from endometrial samples, 4 from ovarian samples, and, 6 from placental samples) generated from 15 pigs by our group in Beijing, China [8,9].

### 2.2. RNA-Seq Data Analyses

First, Trimmomatic (v0.39) [25] was used to trim adaptors and remove low-quality reads. Then, the clean reads were aligned to the Sscrofa11.1 pig reference genome using STAR (2.7.10b) [26]. For the subsequent analysis, 341 samples that had uniquely mapping rates of ≥60% were retained. Finally, the normalized expressions (i.e., Transcripts Per Million, TPM) of 22,838 genes with TPM > 0.1 in >20% of samples using Stringtie (v2.2.1) were obtained [27].

### 2.3. SNP Calling and Quality Control of RNA-Seq Samples

We only considered RNA-seq data from endometrium for eQTL mapping. To identify single nucleotide polymorphisms (SNPs) from RNA-seq samples, PCR duplicates were marked in STAR-aligned BAM files and addressed reads with Ns in their cigar string using the MarkDuplicates and SplitNCigarReads modules from GATK (v4.4.0.0) [28]. The base quality scores were then recalibrated with the GATK BaseRecalibrator and ApplyBQSR modules referencing the dbSNP database (build 150).

Following the best practices for germline variant calling from RNA-seq data, small variants were detected from the recalibrated alignment files, generating individual Genomic Variant Call Format (GVCF) files with the HaplotypeCaller and CombineGVCFs function of GATK. Subsequently, the joint calling of all GVCF samples was performed using the GenotypeGVCFs module from GATK. To extract SNPs from the VCF file while retaining biallelic SNPs, the SelectVariants module was utilized. To select high-quality SNPs, hard filtering was applied with the criteria “FS > 30.0 & QD < 2.0” by the VariantFiltration module. For eQTL mapping, 109,914 SNPs with MAF ≥ 0.05, minor allele count (MAC) ≥ 6, and missing rate of genotypes < 0.2 were considered.

### 2.4. Covariates for eQTL Mapping

Linkage disequilibrium (LD) pruning of the genotypes was initially performed using PLINK (v1.9) [29] with the parameters “--indep-pairwise 200 100 0.1”. Subsequently, principal component analysis (PCA) of samples was then carried out based on the LD-pruned genotypes using PLINK. The top 10 principal components (PCs) were selected as covariates for eQTL mapping. According to the results of the Genotype-Tissue Expression (GTEx) project, for tissues with sample sizes ranging from 250 to 350, 30 PEER factors were used to account for technical confounders among RNA-seq samples. This was achieved using the Probabilistic Estimation of Expression Residuals (PEER) method, implemented in PEER (v1.3) [30] R package. Finally, the gestation days of pigs were included as covariates.

### 2.5. cis-eQTL Mapping and Fine-Mapping

Gene expression values were normalized across samples by the inverse normal transformation. For the eQTL analysis, we focused on SNPs located within 1 Mb upstream and downstream of the transcription start site (TSS) of each gene, which was referred to as cis-eQTL analysis. This analysis was performed using a linear regression model in tensorQTL (v1.0.9) [31] and included the covariates from the previous step.

First, the permutation mode was used to calculate empirical *p*-values with the parameter “--mode cis”. The nominal P-values of cis-eQTL were then obtained with the option of “--mode cis_nominal” of the tensorQTL. The empirical *p*-value corresponding to an FDR threshold of 0.05 was defined as the genome-wide empirical *p*-value threshold. SNPs with nominal P-values below this threshold were considered significant cis-eQTLs. We classified genes with at least one significant cis-eQTL (FDR ≤ 0.05) as eGenes. Significant cis-eQTLs were compared with the Pig QTLdb database from the Animal QTLdb [32].

For fine-mapping analysis of eQTL, the stepwise regression procedure was first employed to map conditionally independent cis-eQTLs by using the tensorQTL with the option of “--mode cis_independent”. Next, fine-mapping of cis-eQTLs for each gene was performed by using the “Sum of Single Effects” (SuSiE) [33] model with the option of “--mode cis_susie” of the tensorQTL. Any SNP with a posterior inclusion probability (PIP) > 0.9 was considered a fine-mapped result. Allelic fold change (aFC) of molQTL was estimated by employing the aFC (v0.3) Python (v3.10) script [34].

### 2.6. WGCNA

TPM from the endometrium, ovary, and placenta was used to construct a gene co-expression network by using the WGCNA package in R [35], and the methods of analysis were based on the study of Xing et al. [36]. First, TPM performed the log_2_(tpm + 1) transformation and then focused on the most variable genes by selecting the top 5000 genes based on the highest median absolute deviation (MAD). A one-step method was then used to construct a network and determine the gene module. The pickSoftThreshold function of the R package WGCNA was used to analyze the expression matrix. The soft thresholding power (β = 7) was confirmed to generate a weighted adjacency matrix. Furthermore, the adjacency matrix can be converted into a topological overlap matrix (TOM), and the corresponding difference degree (1-TOM) can be calculated. The module is divided according to the dynamic TreeCut standard; the minimum capacity of the module is set to 30, and the shear height of the module is set to 0.3 with 1-TOM as the measurement value. The strongest positive correlation was selected for further analysis by calculating the Pearson correlation coefficient between the gene modules and endometrium. Hub genes were screened as potentially endometrial function-related with module membership (MM) > 0.8 and gene significance (GS) > 0.6.

### 2.7. Gene Functional Enrichment Analysis

Gene Ontology (GO) and Kyoto Encyclopedia of Genes and Genomes (KEGG) enrichment analyses of eGenes and hub genes were conducted using the “clusterProfiler” package [37] in R (v4.3.3). The results are visualized using the “ggplot2” package in R.

## 3. Results

### 3.1. cis-eQTL Analysis

**Figure 1:**
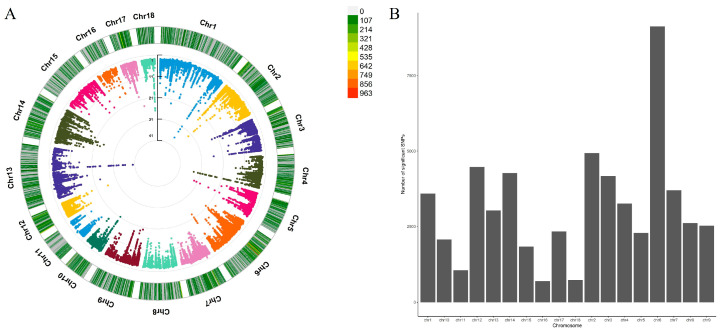
The distribution of significant cis-eQTLs. (**A**) The CMplot of significant cis-eQTLs; chromosomes are shown in different colors; (**B**) number of significant cis-eQTLs on each autosomal chromosome.

After quality control and filtering, 109,914 SNPs and 22,838 genes from the 218 RNA-seq samples were used to search for cis-eQTL in the endometrium. In this study, we identified a total of 34,876 significant cis-eQTLs associated with 5632 eGenes with FDR ≤ 0.05, 6288 independent cis-eQTLs associated with 5632 eGenes, and 1496 fine-mapped cis-eQTLs associated with 1420 eGenes (PIP > 0.9) (Table 1). The distribution of significant cis-eQTLs on autosomes is shown in Figure 1A,B.

**Figure 2:**
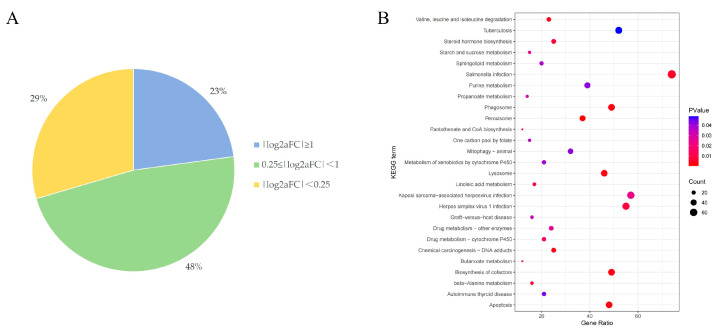
Effect sizes of significant cis-eQTLs and functions of eGenes. (**A**) Percentage of significant cis-eQTLs with different aFC sizes; (**B**) the KEGG pathways of significant eGenes.

aFC was used to reveal the effect size of the eQTL on gene expression levels. In general, an eQTL with |log_2_aFC| ≥ 1 is considered to be eQTL of large effect size, and eQTL with |log_2_aFC| ≤ 0.25 is considered to be eQTL of small effect size [38]. The results showed that 23% of the eQTLs with |log2aFC| ≥ 1, suggesting that these significant cis-eQTLs may have a significant effect on gene expression (Figure 2A).

A search for significant cis-eQTLs at Pig QTLdb revealed that 28 significant cis-eQTLs were annotated to be associated with reproductive traits, of which 10 (rs329485158, rs335299138, rs336670754, rs345476947, rs345895836, rs55618224, rs80829839, rs80972878, rs80986621, and rs80999559) were associated with offspring number, 3 (rs81326570, rs335120977, and rs81474655) with litter weight, 9 (rs341327454, rs80859223, rs81264818, rs81311586, rs81352175, rs81359938, rs81359965, rs692640845, and rs80943493) with teat number, and 6 (rs339238482, rs80919124, rs81215670, rs81217468, rs80978086, and rs80855596) with age at puberty.

To explore the function of significant eGenes in the endometrium, KEGG analysis of significant eGenes enriched a pathway for the biosynthesis of steroid hormones (including estrogen, progesterone, testosterone, etc.), which regulated and influenced several aspects of the reproductive process and other pathways mainly related to metabolism, immune response, and cellular processes, such as Valine, leucine and isoleucine degradation, Phagosome, and Peroxisome (Figure 2B).

### 3.2. Screening of Hub Genes in Endometrium by WGCNA

**Figure 3:**
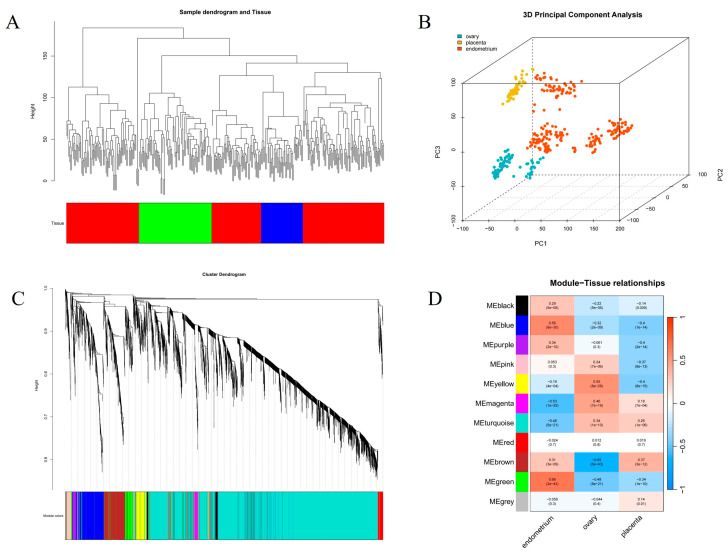
WGCNA in 341 samples. (**A**) Sample clustering by hierarchical clustering: red represents endometrium, green represents ovaries, and blue represents placenta. (**B**) Sample clustering by principal component analysis. (**C**) Gene dendrogram and modules; branches of the same color were divided into the same gene modules. (**D**) Pearson correlation analysis of gene modules and three tissues.

WGCNA was used to identify gene modules and hub genes associated with endometrial function. We used RNA-seq data from the endometrium, ovaries, and placenta to construct the gene expression matrix consisting of 5000 genes from 341 samples after standardized processing. No outlier samples were found in the hierarchical clustering of samples (Figure 3A), and the samples from the three tissues were clustered correctly (Figure 3B).

First, an appropriate soft thresholding power parameter was screened. When the power value was 7, the independence was approximately 0.85, and the average connectedness was relatively high. Then, the correlation matrix and adjacency matrix were calculated and combined into the topology matrix, and a total of 11 gene modules were finally identified (Figure 3C), with dissimilarities less than 0.25 and a minimum module size of 30, based on genetic similarity. The Pearson correlation coefficient between the eigengenes of modules and corresponding variables represented the correlation between the module and phenotypic information. The green and blue modules were positively correlated with the endometrium (r  =  0.66 and r  =  0.56, respectively; *p*  <  0.01) (Figure 3D). These two modules were selected for further analysis.

**Figure 4:**
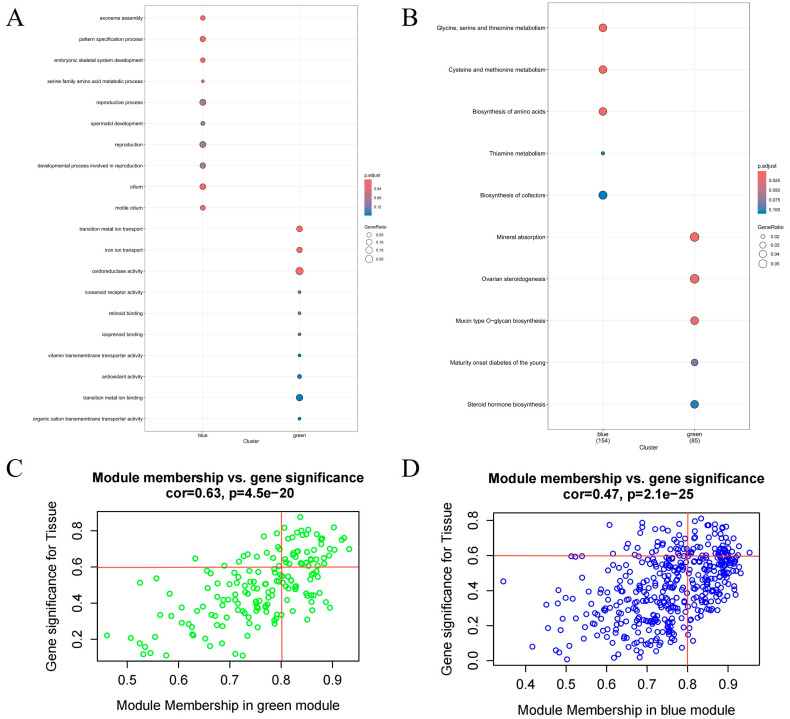
(**A**) GO enrichment analysis for genes in the green and blue modules. (**B**) KEGG enrichment analysis for genes in the green and blue modules. (**C**) Scatterplot of gene significance (GS) for endometrium and module membership (MM) in the green module. (**D**) Scatterplot of GS for endometrium and MM in the blue module.

GO and KEGG analyses were performed for genes in the green and blue modules. GO enrichment analysis showed that the genes in the blue module were mainly enriched in axoneme assembly, pattern specification process, embryonic skeletal system development, reproductive process, reproduction, and cilium, and genes in the green module were enriched in transition metal ion transport, iron ion transport, oxidoreductase activity (Figure 4A). KEGG analysis showed that the genes in the blue module were mainly associated with glycine, serine, and threonine metabolism, biosynthesis of amino acids and biosynthesis of cofactors, and genes in the green module were enriched in mineral absorption, ovarian steroidogenesis, mucin-type O−glycan biosynthesis, and steroid hormone biosynthesis (Figure 4B). The above results indicated that the functions of these two modules were closely related to the reproductive process.

To identify the hub genes associated with the reproductive process in the green and blue modules, scatter plots of GS for endometrium versus MM for the green and blue modules were drawn (Figure 4C,D). A significant correlation between GS and MM was observed in both modules, suggesting that genes within these modules are highly correlated with the reproductive function of the endometrium. Based on the criteria of module membership (MM) > 0.8 and gene significance (GS) > 0.6, 41 hub genes in the green module and 49 hub genes in the blue module were identified, resulting in a total of 90 hub genes associated with endometrial reproductive function across both modules.

### 3.3. Integration of eQTL Analysis and WGCNA Results for Identifying Candidate Genes and cis-eQTLs

**Figure 5:**
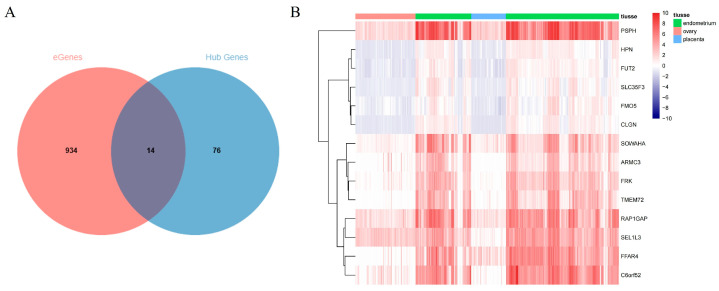
Integration of data from eQTL analysis and WGCNA results. (**A**) Venn map of eGenes associated with fine-mapped eQTLs and hub genes in WGCNA results. (**B**) Heatmap of expression of 14 candidate genes in 341 samples.

To further associate the results of eQTL analysis with reproductive functions in the endometrium, we integrated the results of eQTL and WGCNA analysis. Based on the eQTL analysis, 1420 eGenes and their fine-mapped eQTLs in endometrial tissue were identified. From the WGCNA analysis, 90 hub genes associated with endometrial reproductive functions were identified. Finally, a total of 14 overlapping genes were found, including Fyn Related Src Family Tyrosine Kinase *(FRK)*, Armadillo Repeat Containing 3 (*ARMC3)*, Solute Carrier Family 35 Member F3 (*SLC35F3)*, Transmembrane Protein 72 (*TMEM72)*, Free Fatty Acid Receptor 4 (*FFAR4)*, Sosondowah Ankyrin Repeat Domain Family Member A (*SOWAHA)*, Phosphoserine Phosphatase (*PSPH)*, Flavin Containing Dimethylaniline Monoxygenase 5 (*FMO5)*, Hepsin (*HPN)*, Fucosyltransferase 2 (*FUT2)*, RAP1 GTPase Activating Protein (*RAP1GAP)*, Chromosome 6 Open Reading Frame 52 (*C6orf52)*, SEL1L Family Member 3 (*SEL1L3)*, and Calmegin (*CLGN)* (Figure 5A). These 14 genes exhibited higher expression levels in the endometrium compared to the placenta and ovaries (Figure 5B). Detailed information on the 14 candidate genes and their fine-mapped eQTLs is provided in Table 2.

## 4. Discussion

In this study, we integrated raw RNA-seq data from the public database as well as endometrial RNA-seq data derived from our group for eQTL and WGCNA analysis. Finally, 14 candidate genes potentially associated with endometrial reproductive function and 16 significant fine-mapped eQTLs were identified.

As a reliable method to identify the association between genetic variants and gene expression levels, eQTL mapping can be used to resolve the regulatory mechanisms of genetic variants on gene expression, as well as to provide insights into the mechanisms underlying complex phenotypes of GWAS [39,40]. Similar to GWAS analysis, eQTL analysis has sample size requirements, with a minimum of 60 samples required due to the limitations of biological sample availability and the costs associated with sample collection and molecular testing, a few hundred samples being sufficient for standard cis-eQTL analysis, and 200–300 samples being sufficient for identification of stable eQTLs of large effect [41,42]. In our study, we only focused on cis-eQTL analysis. Trans-eQTLs are located distally at genes (>5 Mb) or on different chromosomes and typically have smaller effect sizes than cis-eQTLs, thus requiring larger sample sizes for detection [43]. Endometrial tissue plays an important role in the estrus and gestation process of sows, which is significant for improving their reproductive performance. However, obtaining endometrial samples is an invasive procedure that may incur economic losses, making it difficult to obtain samples in large quantities. Therefore, we adopted the analysis methods from the Genotype-Tissue Expression (GTEx) project and the Farm Animal Genotype-Tissue Expression (FarmGTEx) project, leveraging public databases for analysis.

In our study, 34,876 significant cis-eQTLs associated with r eGenes were identified. The effect size of an eQTL describes the magnitude of its impact on gene expression. Log allelic fold change (aFC), which represents the magnitude of expression change associated with a given genetic variant, is a reliable measure for quantifying the effect size of cis-eQTLs and serves as a biologically interpretable unit [34]. In our results, 23% of the significant cis-eQTLs with |log2aFC| ≥ 1 were considered as cis-eQTLs with a large effect, indicating that these loci have a large effect on gene expression levels. The limitation of sample size resulted in the detection of fewer cis-eQTLs with a small effect [23,38]. The existence of linkage disequilibrium between different loci leads to the identification of causal variants that truly affect the phenotype becomes challenging. In this study, we employed fine-mapping approaches to identify causal genetic variants that influence gene expression levels [44], 1496 fine-mapped cis-eQTLs that were significantly associated with 1420 eGenes were identified, which included 1 to 4 fine-mapped eQTLs for each eGene.

The KEGG functional enrichment analysis revealed that significant eGenes were enriched in pathways related to hormone regulation, metabolism, and immune response. The pathways of steroid hormone biosynthesis, Valine, leucine, and isoleucine degradation, Sphingolipid metabolism, and Peroxisome are associated with reproductive functions. Steroid hormones, amino acids, and lipids play an important role in regulating the cyclical changes of the uterus and preparing the uterine environment for embryo implantation and pregnancy maintenance [45,46,47,48]. Higher expression of antioxidant enzymes in decidual cells protects the embryo from the effects of endometrial oxidative stress generated during the implantation process [49].

In this study, two gene modules related to endometrial reproductive function and 90 hub genes were identified through WGCNA, including the major gene *RBP4* associated with litter size [50]. WGCNA clusters genes with similar functions or signaling pathways into a module, and the interactions between these genes can be revealed at the system level, helping researchers to gain deeper insights into the mechanisms underlying gene interactions [36]. By overlapping eGenes with hub genes, 14 candidate genes associated with endometrial reproductive function were identified, including *FRK*, *ARMC3*, *SLC35F3*, *TMEM72*, *FFAR4*, *SOWAHA*, *PSPH*, *FMO5, HPN*, *FUT2*, *RAP1GAP*, *C6orf52*, *SEL1L3,* and *CLGN*. These genes exhibited higher expression levels in endometrial tissues compared to ovarian and placental tissues, suggesting that these eQTLs might regulate reproductive physiological functions by increasing the expression of the candidate genes.

*FUT2* and *FFAR4* are directly involved in the embryo implantation process. The *FUT2* gene encodes fucosyltransferase 2, which catalyzes the transfer of fucose in α1,2 linkages to generate H-type and Lewis antigens. This process is implicated in the acquisition of receptivity for embryo attachment and contributes to maternal-fetal recognition [51]. Wu et al. [52] demonstrated that reduced expression of *FUT2* in the duodenum decreased the adhesion of enterotoxigenic Escherichia coli F18 to small intestinal epithelial cells, thereby reducing porcine post-weaning diarrhea and improving piglet survival rates, which suggested that *FUT2* was involved in cell adhesion. Wang et al. [53] identified a significant association between rs345476947 in the *FUT2* intron and the number of newborn piglets and weaned piglets through a GWAS study, and individuals with the TT genotype had significantly higher numbers of newborn piglets and weaned piglets than those with the CC genotype. In our study, two newly fine-mapped cis-eQTLs (rs323859419, rs327066479) were significantly associated with the expression level of *FUT2*. Further investigation is required to explore the regulatory mechanisms of these loci for *FUT2*. Free-fatty acid receptor-4 (*FFAR4*), also known as *GPR120*, plays a key role in the regulation of **ω**-3 fatty acids and glucose metabolism. Huang et al. [54] demonstrated that *GPR120* promoted decidualization by upregulating glucose uptake and the pentose-phosphate pathway (PPP) in human endometrial stromal cells, exerting a protective role in maintaining pregnancy.

*FRK*, *RAP1GAP*, *ARMC3*, and *CLGN* are associated with cell adhesion, motile cilia, and protein folding. Fyn-related src family tyrosine kinase (FRK), which is also known as protein tyrosine kinase 5 (PTK5) or Rak, is a member of the SRC family kinases. Tyrosine kinases are critical regulators of various cellular processes, including cell migration, differentiation, cell–cell adhesion, exocrine signaling, proliferation, and death. Shi et al. [55] reported that *FRK* over-expression increased the protein level of N-cadherin. The *RAP1GAP* gene encodes a protein that exhibits GTPase-activating protein (GAP) activity specific to Rap1 and plays a role in cell adhesion processes. A previous study ha demonstrated that the depletion of *Rap1GAP* resulted in a reduced accumulation of E-cadherin and **β**-catenin at the cell–cell junctions [56]. Cell–cell adhesion molecules are critically involved in the early events of reproduction, which include gamete transport, fertilization, embryonic development, and implantation [57]. Armadillo repeat-containing proteins (ARMCs) comprise a large family that plays an important role in regulating cytoskeletal function, mitochondrial function, tumorigenesis, intracellular signaling, and cell–cell adhesion [58]. A previous study indicated that the *ARMC3* gene was associated with human motile cilia [59]. The endometrial epithelium consists of secretory and ciliated cells, which may play a role in transporting secretory substances from glandular and tubular epithelial cells into the uterine cavity [60]. Calmegin (*CLGN*) encodes a calcium-binding protein localized on the endoplasmic reticulum membrane, homologous to calnexin. In somatic cells, calnexin associates transiently with nascent membranes and soluble glycoproteins in the secretory pathway within the endoplasmic reticulum, facilitating protein folding and ensuring normal cellular function [61].

*SLC35F3* and *PSPH* are involved in vitamin and amino acid metabolism. *SLC35F3* gene encodes solute carrier family 35 member F3, which may transport thiamine, a water-soluble vitamin of the B complex (vitamin B1), playing a crucial role in cellular glucose metabolism [62]. The *PSPH* gene encodes a phosphoserine phosphatase belonging to the haloacid dehalogenase superfamily, which plays an important role in L-serine biosynthesis and influences cell proliferation and differentiation. The expression of the *PSPH* gene is regulated by estrogen [63].

*FMO5* and *SOWAHA* are regulated by sex hormones. The promoter of flavin-containing monooxygenase 5 (*FMO5*) contains progesterone/glucocorticoid response elements and is regulated by progesterone levels [64], which are primarily involved in drug metabolism in the liver [65]. The high expression of *FMO5* in the endometrium may be induced by elevated progesterone levels in the uterus. The expression of *SOWAHA* was reported to be affected by follicle stimulation hormone (FSH) in granulosa cells of ovarian follicles [66].

*HPN*, *TMEM72*, and *SEL1L3* play an important role in cell membrane function, protein transport, and stress response. The hepsin gene (*HPN*) encodes a type II transmembrane serine protease. The functions of this protease include regulating epithelial integrity and the proteolytic activation of hepatocyte growth factor (HGF) and macrophage-stimulating protein (MSP), and it may be involved in embryo implantation [67]. The transmembrane protein (TMEM) family is a group of proteins that integrate with cellular biological membranes. TMEMs play essential roles in several cellular processes, such as the transport of materials across membranes, energy conversion, signal transduction, and the maintenance of membrane homeostasis. The specific member of this family, TMEM72, is involved in protein-membrane trafficking [68]. The *SEL1L3* gene encodes the SEL1L (Sel-1 Suppressor of Lin-12-Like) family member 3 protein, located in the endoplasmic reticulum, which is involved in the regulation of cellular stress homeostasis [69].

Unfortunately, our study lacked reproductive phenotype data, preventing us from directly linking variates–gene–phenotype relationships through association analysis. However, cis-eQTLs related to endometrial function were revealed by eQTL analysis. By combining eQTL analysis with WGCNA, we were still able to identify candidate genes and eQTLs associated with reproductive functions. These genes and eQTLs may help us to understand the underlying mechanisms regulating reproductive functions.

## 5. Conclusions

Our study provides novel insights into the genetic regulation of reproductive traits in pigs by integrating eQTL mapping and WGCNA. We identified 14 candidate genes and 16 fine-mapped cis-eQTLs associated with endometrial reproductive function, highlighting their potential roles in hormonal regulation, cell adhesion, and metabolic processes critical for reproduction. These fine-mapped cis-eQTLs regulate the high expression of candidate genes in the endometrium, thereby affecting reproductive-related physiological functions. These findings not only enhance our understanding of genetic regulatory mechanisms in the endometrium but also provide functional genetic markers for precision breeding and marker-assisted selection (MAS) in pigs. By elucidating the molecular mechanisms underlying reproductive functions, our study lays the foundation for future genetic improvement strategies aimed at increasing sow fertility and optimizing breeding efficiency.

## Figures and Tables

**Table 1:** Summary of the number of cis-eQTLs and eGenes.

Type	Significant	Independent	Fine Mapped
eGene	5632	5632	1420
eQTL	34,876	6288	1496
eGene-eQTL	56,660	6677	1549

**Table 2:** Summary of candidate genes and fine-mapped cis-eQTLs.

Ensemble ID	Gene	eQTL Position	RS Number	PIP	Pval_Nominal
ENSSSCG00000004434	FRK	1_81907491	rs333994665	0.9962785	6.31 × 10^−6^
ENSSSCG00000011078	ARMC3	10_52338433	rs319228529	0.93160963	1.01 × 10^−4^
ENSSSCG00000010162	SLC35F3	14_56740841	rs338283666	0.955412	1.02 × 10^−4^
ENSSSCG00000010162	SLC35F3	14_57972209	rs324855920	0.9711524	1.53 × 10^−4^
ENSSSCG00000025787	TMEM72	14_90784347	rs335313886	0.97402775	8.00 × 10^−4^
ENSSSCG00000010478	FFAR4	14_104040504	rs341695379	0.9788259	2.10 × 10^−8^
ENSSSCG00000014286	SOWAHA	2_135149975	rs81365495	0.9513308	1.41 × 10^−6^
ENSSSCG00000007748	PSPH	3_16858682	rs1109047347	0.97025245	1.75 × 10^−7^
ENSSSCG00000006702	FMO5	4_100371933	rs336496720	0.9379143	5.29 × 10^−5^
ENSSSCG00000021867	HPN	6_44606729	rs692754439	0.9981295	4.59 × 10^−10^
ENSSSCG00000003145	FUT2	6_54046253	rs323859419	0.97183824	1.16 × 10^−4^
ENSSSCG00000003145	FUT2	6_53069250	rs327066479	0.97199005	2.44 × 10^−4^
ENSSSCG00000022029	RAP1GAP	6_80017044	rs55619266	0.9862745	2.21 × 10^−6^
ENSSSCG00000001040	C6orf52	7_7455876	rs80974566	0.9836499	8.59 × 10^−10^
ENSSSCG00000022446	SEL1L3	8_19714479	rs320263410	0.9991525	1.04 × 10^−5^
ENSSSCG00000026360	CLGN	8_87641656	rs343689578	0.98143643	1.69 × 10^−5^

## Data Availability

All raw data from public databases analyzed in this study can be downloaded from SRA (https://www.ncbi.nlm.nih.gov/sra/, accessed on 5 April 2024) and ENA (https://www.ebi.ac.uk/ena/browser/home, 5 April 2024). The dataset from our group in this study is available from the corresponding author upon reasonable request.

## Supplementary Materials

The following supporting information can be downloaded at: https://www.mdpi.com/article/10.3390/ani15071038/s1, Table S1: Detailed information on RNA-seq data for 341 samples.

## References

[1] Benjamin M., Yik S. (2019). Precision Livestock Farming in Swine Welfare: A Review for Swine Practitioners. Animals.

[2] Nonneman D.J., Lents C.A. (2023). Functional Genomics of Reproduction in Pigs: Are We There Yet?. Mol. Reprod. Dev..

[3] Zak L.J., Gaustad A.H., Bolarin A., Broekhuijse M.L.W.J., Walling G.A., Knol E.F. (2017). Genetic Control of Complex Traits, with a Focus on Reproduction in Pigs. Mol. Reprod. Dev..

[4] Spötter A., Distl O. (2006). Genetic Approaches to the Improvement of Fertility Traits in the Pig. Vet. J..

[5] Yin C., Zhou P., Wang Y., Yin Z., Liu Y. (2024). Using Genomic Selection to Improve the Accuracy of Genomic Prediction for Multi-Populations in Pigs. Animal.

[6] Almeida F.R.C.L., Dias A.L.N.A. (2022). Pregnancy in Pigs: The Journey of an Early Life. Domest. Anim. Endocrinol..

[7] Kridli R.T., Khalaj K., Bidarimath M., Tayade C. (2016). Placentation, Maternal–Fetal Interface, and Conceptus Loss in Swine. Theriogenology.

[8] Zhang H., Liu Z., Wang J., Zeng T., Ai X., Wu K. (2023). An Integrative ATAC-Seq and RNA-Seq Analysis of the Endometrial Tissues of Meishan and Duroc Pigs. Int. J. Mol. Sci..

[9] Li W.-T., Zhang M.-M., Li Q.-G., Tang H., Zhang L.-F., Wang K.-J., Zhu M.-Z., Lu Y.-F., Bao H.-G., Zhang Y.-M. (2017). Whole-Genome Resequencing Reveals Candidate Mutations for Pig Prolificacy. Proc. R. Soc. B Biol. Sci..

[10] Liu Z., Xu R., Zhang H., Wang D., Wang J., Wu K. (2022). A Unique 15-Bp InDel in the First Intron of BMPR1B Regulates Its Expression in Taihu Pigs. BMC Genom..

[11] Liu Z., Zhang H., Wang J., Wang D., Zeng T., Ai X., Wang X., Zhao X., Wu K. (2025). Functional Effects of *BMPR1B* in Porcine Endometrium Provides Novel Insights into the High Fecundity of Taihu Pigs. Int. J. Biol. Macromol..

[12] Xing L., Lu X., Zhang W., Wang Q., Zhang W. (2024). Genetic Structure and Genome-Wide Association Analysis of Growth and Reproductive Traits in Fengjing Pigs. Animals.

[13] Zhao Y.X., Gao G.X., Zhou Y., Guo C.X., Li B., El-Ashram S., Li Z.L. (2022). Genome-Wide Association Studies Uncover Genes Associated with Litter Traits in the Pig. Animal.

[14] Zhang Z., Chen Z., Ye S., He Y., Huang S., Yuan X., Chen Z., Zhang H., Li J. (2019). Genome-Wide Association Study for Reproductive Traits in a Duroc Pig Population. Animals.

[15] Schipper M., Posthuma D. (2022). Demystifying Non-Coding GWAS Variants: An Overview of Computational Tools and Methods. Hum. Mol. Genet..

[16] Flynn E.D., Lappalainen T. (2022). Functional Characterization of Genetic Variant Effects on Expression. Annu. Rev. Biomed. Data Sci..

[17] Pai A.A., Pritchard J.K., Gilad Y. (2015). The Genetic and Mechanistic Basis for Variation in Gene Regulation. PLoS Genet..

[18] Safaei S.M.H., Dadpasand M., Mohammadabadi M., Atashi H., Stavetska R., Klopenko N., Kalashnyk O. (2023). An *Origanum majorana* Leaf Diet Influences Myogenin Gene Expression, Performance, and Carcass Characteristics in Lambs. Animals.

[19] Mohammadabadi M.R., Torabi A., Tahmourespoor M., Baghizadeh A., Koshkoie A.E., Mohammadi A. (2010). Analysis of Bovine Growth Hormone Gene Polymorphism of Local and Holstein Cattle Breeds in Kerman Province of Iran Using Polymerase Chain Reaction Restriction Fragment Length Polymorphism (PCR-RFLP). Afr. J. Biotechnol..

[20] Cai W., Hu J., Zhang Y., Guo Z., Zhou Z., Hou S. (2024). Cis-eQTLs in Seven Duck Tissues Identify Novel Candidate Genes for Growth and Carcass Traits. BMC Genom..

[21] Cai W., Zhang Y., Chang T., Wang Z., Zhu B., Chen Y., Gao X., Xu L., Zhang L., Gao H. (2023). The eQTL Colocalization and Transcriptome-Wide Association Study Identify Potentially Causal Genes Responsible for Economic Traits in Simmental Beef Cattle. J. Anim. Sci. Biotechnol..

[22] Drag M.H., Kogelman L.J.A., Maribo H., Meinert L., Thomsen P.D., Kadarmideen H.N. (2019). Characterization of eQTLs Associated with Androstenone by RNA Sequencing in Porcine Testis. Physiol. Genom..

[23] Aguet F., Anand S., Ardlie K.G., Gabriel S., Getz G.A., Graubert A., Hadley K., Handsaker R.E., Huang K.H., The GTEx Consortium (2020). The GTEx Consortium Atlas of Genetic Regulatory Effects across Human Tissues. Science.

[24] Teng J., Gao Y., Yin H., Bai Z., Liu S., Zeng H., Bai L., Cai Z., Zhao B., Li X. (2024). A Compendium of Genetic Regulatory Effects across Pig Tissues. Nat. Genet..

[25] Bolger A.M., Lohse M., Usadel B. (2014). Trimmomatic: A Flexible Trimmer for Illumina Sequence Data. Bioinformatics.

[26] Dobin A., Davis C.A., Schlesinger F., Drenkow J., Zaleski C., Jha S., Batut P., Chaisson M., Gingeras T.R. (2013). STAR: Ultrafast Universal RNA-Seq Aligner. Bioinformatics.

[27] Pertea M., Pertea G.M., Antonescu C.M., Chang T.-C., Mendell J.T., Salzberg S.L. (2015). StringTie Enables Improved Reconstruction of a Transcriptome from RNA-Seq Reads. Nat. Biotechnol..

[28] McKenna A., Hanna M., Banks E., Sivachenko A., Cibulskis K., Kernytsky A., Garimella K., Altshuler D., Gabriel S., Daly M. (2010). The Genome Analysis Toolkit: A MapReduce Framework for Analyzing next-Generation DNA Sequencing Data. Genome Res..

[29] Chang C.C., Chow C.C., Tellier L.C., Vattikuti S., Purcell S.M., Lee J.J. (2015). Second-Generation PLINK: Rising to the Challenge of Larger and Richer Datasets. GigaScience.

[30] Stegle O., Parts L., Piipari M., Winn J., Durbin R. (2012). Using Probabilistic Estimation of Expression Residuals (PEER) to Obtain Increased Power and Interpretability of Gene Expression Analyses. Nat. Protoc..

[31] Taylor-Weiner A., Aguet F., Haradhvala N.J., Gosai S., Anand S., Kim J., Ardlie K., Van Allen E.M., Getz G. (2019). Scaling Computational Genomics to Millions of Individuals with GPUs. Genome Biol..

[32] Hu Z.-L., Park C.A., Reecy J.M. (2022). Bringing the Animal QTLdb and CorrDB into the Future: Meeting New Challenges and Providing Updated Services. Nucleic Acids Res..

[33] Zhang X., Jiang W., Zhao H. (2024). Integration of Expression QTLs with Fine Mapping via SuSiE. PLoS Genet..

[34] Mohammadi P., Castel S.E., Brown A.A., Lappalainen T. (2017). Quantifying the Regulatory Effect Size of Cis-Acting Genetic Variation Using Allelic Fold Change. Genome Res..

[35] Langfelder P., Horvath S. (2008). WGCNA: An R Package for Weighted Correlation Network Analysis. BMC Bioinform..

[36] Xing K., Liu H., Zhang F., Liu Y., Shi Y., Ding X., Wang C. (2021). Identification of Key Genes Affecting Porcine Fat Deposition Based on Co-Expression Network Analysis of Weighted Genes. J. Anim. Sci. Biotechnol..

[37] Wu T., Hu E., Xu S., Chen M., Guo P., Dai Z., Feng T., Zhou L., Tang W., Zhan L. (2021). clusterProfiler 4.0: A Universal Enrichment Tool for Interpreting Omics Data. Innovation.

[38] Guan D., Bai Z., Zhu X., Zhong C., Hou Y., Lan F., Diao S., Yao Y., Zhao B., Zhu D. (2023). The ChickenGTEx Pilot Analysis: A Reference of Regulatory Variants across 28 Chicken Tissues. bioRxiv.

[39] Tang Y., Zhang J., Li W., Liu X., Chen S., Mi S., Yang J., Teng J., Fang L., Yu Y. (2024). Identification and Characterization of Whole Blood Gene Expression and Splicing Quantitative Trait Loci during Early to Mid-Lactation of Dairy Cattle. BMC Genom..

[40] Diniz W.J.S., Afonso J., Kertz N.C., Dyce P.W., Banerjee P. (2024). Mapping Expression Quantitative Trait Loci Targeting Candidate Genes for Pregnancy in Beef Cows. Biomolecules.

[41] Tam V., Patel N., Turcotte M., Bossé Y., Paré G., Meyre D. (2019). Benefits and Limitations of Genome-Wide Association Studies. Nat. Rev. Genet..

[42] Aguet F., Alasoo K., Li Y.I., Battle A., Im H.K., Montgomery S.B., Lappalainen T. (2023). Molecular Quantitative Trait Loci. Nat. Rev. Methods Primers.

[43] Võsa U., Claringbould A., Westra H.-J., Bonder M.J., Deelen P., Zeng B., Kirsten H., Saha A., Kreuzhuber R., Yazar S. (2021). Large-Scale Cis- and Trans-eQTL Analyses Identify Thousands of Genetic Loci and Polygenic Scores That Regulate Blood Gene Expression. Nat. Genet..

[44] Schaid D.J., Chen W., Larson N.B. (2018). From Genome-Wide Associations to Candidate Causal Variants by Statistical Fine-Mapping. Nat. Rev. Genet..

[45] Kim J.-M., Park J.-E., Yoo I., Han J., Kim N., Lim W.-J., Cho E.-S., Choi B., Choi S., Kim T.-H. (2018). Integrated Transcriptomes throughout Swine Oestrous Cycle Reveal Dynamic Changes in Reproductive Tissues Interacting Networks. Sci. Rep..

[46] Forde N., Simintiras C.A., Sturmey R., Mamo S., Kelly A.K., Spencer T.E., Bazer F.W., Lonergan P. (2014). Amino Acids in the Uterine Luminal Fluid Reflects the Temporal Changes in Transporter Expression in the Endometrium and Conceptus during Early Pregnancy in Cattle. PLoS ONE.

[47] Jia Z., Wei Y., Zhang Y., Song K., Yuan J. (2024). Metabolic Reprogramming and Heterogeneity during the Decidualization Process of Endometrial Stromal Cells. Cell Commun. Signal..

[48] Yang T., Zhao J., Liu F., Li Y. (2022). Lipid Metabolism and Endometrial Receptivity. Hum. Reprod. Update.

[49] Artimovič P., Badovská Z., Toporcerová S., Špaková I., Smolko L., Sabolová G., Kriváková E., Rabajdová M. (2024). Oxidative Stress and the Nrf2/PPARγ Axis in the Endometrium: Insights into Female Fertility. Cells.

[50] Vaishnav S., Chauhan A., Ajay A., Saini B.L., Kumar S., Kumar A., Bhushan B., Gaur G.K. (2023). Allelic to Genome Wide Perspectives of Swine Genetic Variation to Litter Size and Its Component Traits. Mol. Biol. Rep..

[51] Nakamura H., Jasper M.J., Hull M.L., Aplin J.D., Robertson S.A. (2012). Macrophages Regulate Expression of A1,2-Fucosyltransferase Genes in Human Endometrial Epithelial Cells. Mol. Hum. Reprod..

[52] Wu Z., Feng H., Cao Y., Huang Y., Dai C., Wu S., Bao W. (2018). New Insight into the Molecular Mechanism of the FUT2 Regulating *Escherichia coli* F18 Resistance in Weaned Piglets. Int. J. Mol. Sci..

[53] Wang H., Wu S., Wu J., Sun S., Wu S., Bao W. (2018). Association Analysis of the SNP (Rs345476947) in the *FUT2* Gene with the Production and Reproductive Traits in Pigs. Genes Genom..

[54] Huang J., Xue M., Zhang J., Yu H., Gu Y., Du M., Ye W., Wan B., Jin M., Zhang Y. (2019). Protective Role of GPR120 in the Maintenance of Pregnancy by Promoting Decidualization via Regulation of Glucose Metabolism. EBioMedicine.

[55] Shi Q., Song X., Wang J., Gu J., Zhang W., Hu J., Zhou X., Yu R. (2015). FRK Inhibits Migration and Invasion of Human Glioma Cells by Promoting N-Cadherin/β-Catenin Complex Formation. J. Mol. Neurosci..

[56] Tamate M., Tanaka R., Osogami H., Matsuura M., Satohisa S., Iwasaki M., Saito T. (2017). Rap1GAP Inhibits Tumor Progression in Endometrial Cancer. Biochem. Biophys. Res. Commun..

[57] D’Occhio M.J., Campanile G., Zicarelli L., Visintin J.A., Baruselli P.S. (2020). Adhesion Molecules in Gamete Transport, Fertilization, Early Embryonic Development, and Implantation—Role in Establishing a Pregnancy in Cattle: A Review. Mol. Reprod. Dev..

[58] Huang Y., Jiang Z., Gao X., Luo P., Jiang X. (2021). ARMC Subfamily: Structures, Functions, Evolutions, Interactions, and Diseases. Front. Mol. Biosci..

[59] Patir A., Fraser A.M., Barnett M.W., McTeir L., Rainger J., Davey M.G., Freeman T.C. (2020). The Transcriptional Signature Associated with Human Motile Cilia. Sci. Rep..

[60] Spassky N., Meunier A. (2017). The Development and Functions of Multiciliated Epithelia. Nat. Rev. Mol. Cell Biol..

[61] Ikawa M., Wada I., Kominami K., Watanabe D., Toshimori K., Nishimune Y., Okabe M. (1997). The Putative Chaperone Calmegin Is Required for Sperm Fertility. Nature.

[62] Zang X.-L., Han W.-Q., Yang F.-P., Ji K.-D., Wang J.-G., Gao P.-J., He G., Wu S.-N. (2016). Association of a SNP in SLC35F3 Gene with the Risk of Hypertension in a Chinese Han Population. Front. Genet..

[63] Lee J.-Y., Lim W., Jo G., Bazer F.W., Song G. (2015). Estrogen Regulation of Phosphoserine Phosphatase during Regression and Recrudescence of Female Reproductive Organs. Gen. Comp. Endocrinol..

[64] Uno Y., Shimizu M., Ogawa Y., Makiguchi M., Kawaguchi H., Yamato O., Ishizuka M., Yamazaki H. (2022). Molecular and Functional Characterization of Flavin-Containing Monooxygenases in Pigs, Dogs, and Cats. Biochem. Pharmacol..

[65] Miller M.M., James R.A., Richer J.K., Gordon D.F., Wood W.M., Horwitz K.B. (1997). Progesterone Regulated Expression of Flavin-Containing Monooxygenase 5 by the B-Isoform of Progesterone Receptors: Implications for Tamoxifen Carcinogenicity. J. Clin. Endocrinol. Metab..

[66] Zhong C., Liu Z., Qiao X., Kang L., Sun Y., Jiang Y. (2020). Integrated Transcriptomic Analysis on Small Yellow Follicles Reveals That Sosondowah Ankyrin Repeat Domain Family Member A Inhibits Chicken Follicle Selection. Anim. Biosci..

[67] Belitškin D., Munne P., Pant S.M., Anttila J.M., Suleymanova I., Belitškina K., Kirchhofer D., Janetka J., Käsper T., Jalil S. (2024). Hepsin Promotes Breast Tumor Growth Signaling via the TGFβ-EGFR Axis. Mol. Oncol..

[68] Ding J., Matsumiya T., Miki Y., Hayakari R., Shiba Y., Kawaguchi S., Seya K., Imaizumi T. (2023). ER Export Signals Mediate Plasma Membrane Localization of Transmembrane Protein TMEM72. FEBS J..

[69] Shen C.-Y., Chang W.-H., Chen Y.-J., Weng C.-W., Regmi P., Kier M.K.K., Su K.-Y., Chang G.-C., Chen J.-S., Chen Y.-J. (2023). Tissue Proteogenomic Landscape Reveals the Role of Uncharacterized SEL1L3 in Progression and Immunotherapy Response in Lung Adenocarcinoma. J. Proteome Res..

